# Metabolic licensing and restriction of innate immunity in the tumor microenvironment

**DOI:** 10.3389/fimmu.2026.1935435

**Published:** 2026-09-16

**Authors:** Ji Min Shin, Hye Yeon Choi, Jihyoun Kim, Seon Ah Lim

**Affiliations:** 1 Department of Life Science, Ewha Womans University, Seoul, Republic of Korea; 2 Research Center for Cellular Homeostasis, Ewha Womans University, Seoul, Republic of Korea; 3 Multitasking Macrophage Research Center, Ewha Womans University, Seoul, Republic of Korea

**Keywords:** immunometabolism, innate immunity, metabolic licensing, metabolic restriction, metabolic stress, tumor microenvironment

## Abstract

Tumor progression is driven by metabolic remodeling that generates a microenvironment characterized by nutrient deprivation, hypoxia, lactate accumulation, lipid dysregulation, and oxidative stress. These conditions affect innate immune populations, including natural killer (NK) cells, dendritic cells (DCs), macrophages, neutrophils, and myeloid-derived suppressor cells (MDSCs), shaping their activation, persistence, and functional states. Although many studies have defined metabolic pathways that regulate innate immune function in cancer, these findings are often discussed at the level of individual pathways or individual cell types, obscuring shared principles by which the tumor microenvironment controls innate immunity. Here, we use the concepts of metabolic licensing and metabolic restriction to describe how metabolic capacity and environmental constraints interact to shape innate immune function. Metabolic licensing refers to context-dependent metabolic states that provide sufficient bioenergetic and biosynthetic capacity to support sustained antitumor effector function, whereas metabolic restriction describes conditions in which nutrient limitation, mitochondrial dysfunction, redox imbalance, or suppressive metabolites constrain or progressively erode these functions. Rather than representing fixed binary states, licensing and restriction can occur along a continuum shaped by cell identity, signal duration, and local tumor conditions. We discuss how metabolic licensing and restriction shape antitumor and immunosuppressive innate immune populations, examine the stress-sensing pathways that connect environmental cues to innate immune fate, and summarize therapeutic strategies aimed at restoring metabolic fitness or alleviating metabolic restriction. Considering innate immune responses in terms of metabolic licensing and restriction helps explain how shared metabolic pressures within tumors can impair effector cells while supporting suppressive innate populations and may inform the development of immunometabolic approaches to cancer therapy.

## 1 Introduction

Solid tumors arise within a metabolically stressed microenvironment shaped by uncontrolled proliferation, inadequate vascularization, and chronic inflammation. As tumors expand, increasing demands for nutrients and oxygen alter local metabolic conditions, leading to glucose depletion, amino acid scarcity, hypoxia, lactate accumulation, oxidative stress, and changes in lipid availability (1, 2). Once regarded mainly as consequences of tumor growth, these metabolic alterations are now understood to influence the behavior of cancer cells as well as surrounding stromal and immune populations. Metabolic stress therefore contributes not only to tumor progression and immune evasion but also to therapeutic responsiveness (3).

Immunometabolism has shown that metabolic pathways do more than provide energy and biosynthetic substrates for immune cells (4). They also influence activation, differentiation, effector function, and persistence. Most studies in cancer have focused on T cells, but innate immune populations are equally exposed to the metabolic conditions of the tumor microenvironment (TME). Natural killer (NK) cells, dendritic cells (DCs), macrophages, neutrophils, and myeloid-derived suppressor cells (MDSCs) encounter continuous changes in nutrient availability, oxygen tension, and inflammatory signals (5, 6). Their responses to these changes are closely tied to their metabolic state.

Activation of innate immune cells is accompanied by extensive metabolic remodeling. Upon stimulation, NK cells and DCs rapidly increase glycolytic activity, alter mitochondrial function, and adjust amino acid utilization to support cytotoxicity, cytokine production, antigen presentation, and inflammatory responses (7, 8). Similarly, inflammatory macrophages undergo distinct metabolic transitions that facilitate antimicrobial and antitumor functions (7). These observations suggest that metabolic pathways do not merely provide fuel for activated immune cells. Rather, specific metabolic programs can provide the bioenergetic and biosynthetic capacity required for innate immune activation and sustained function.

In contrast, prolonged exposure to metabolic stress within tumors can impair innate immune fitness and compromise antitumor immunity (9, 10). Competition for nutrients, accumulation of immunosuppressive metabolites such as lactate, chronic hypoxia, mitochondrial dysfunction, and excessive reactive oxygen species (ROS) collectively disrupt immune effector programs (9, 11). Importantly, these conditions do not simply inhibit immune activity. Chronic metabolic stress can progressively reshape innate immune cell states and contribute to dysfunction, tolerance, or immunosuppressive reprogramming (12, 13). Furthermore, the TME can favor the persistence of suppressive innate populations, including tumor-associated macrophages (TAMs), MDSCs, and tumor-associated neutrophils (TANs), which exploit distinct metabolic adaptations to survive and function under hostile conditions (14).

Although substantial progress has been made in defining metabolic pathways that regulate individual innate immune populations, common principles governing how metabolic conditions shape innate immune fate have received less attention. Across diverse innate immune cell types, similar metabolic pressures repeatedly influence activation, dysfunction, and suppressive reprogramming. Here, we use the terms metabolic licensing and metabolic restriction to describe how metabolic capacity and environmental constraints influence the ability of innate immune cells to execute and sustain antitumor function.

Metabolic licensing refers to a context-dependent state in which nutrient availability, glycolytic capacity, mitochondrial fitness, and metabolic flexibility are sufficient to support sustained antitumor effector function in response to activating signals. In contrast, metabolic restriction describes conditions in which nutrient limitation, mitochondrial dysfunction, redox imbalance, or suppressive metabolites constrain or progressively erode these functions. We do not view licensing and restriction as fixed binary states; rather, they can represent reversible positions along a continuum shaped by cell identity, signal duration, spatial location within the tumor, disease stage, and prior activation history.

In this review, we discuss how metabolic stress shapes innate immune responses across diverse cellular populations within the TME. We first examine the metabolic programs that license effective antitumor innate immunity and then explore the mechanisms through which metabolic stress restricts immune function. We further discuss how suppressive innate populations adapt to metabolically stressed tumor niches, highlight shared metabolic stress-sensing pathways that integrate environmental cues with immune cell fate decisions, and summarize therapeutic strategies aimed at restoring innate immune metabolic fitness. Together, these perspectives clarify how metabolic stress shapes innate immune fate and may guide therapeutic intervention in cancer.

## 2 Metabolic licensing of antitumor innate immunity

### 2.1 Concept of metabolic licensing

Metabolic regulation of glycolysis, oxidative metabolism, and glutaminolysis fuels cell fate decisions and effector functions of immune cells in response to extracellular signals (15). Immune cells exhibit considerable flexibility in how they engage these pathways, switching between glycolysis, oxidative phosphorylation, and anaplerotic routes depending on nutrient availability and inflammatory cues. This flexibility contributes to whether innate immune cells can maintain activation and antitumor effector programs under stress, such that metabolism functions as a licensing system for immune activation (16).

We use the term metabolic licensing to describe metabolic states that actively permit innate immune cells to acquire and sustain antitumor effector functions. In this view, metabolic programs do not simply provide ATP or biosynthetic building blocks; they operate as regulatory modules that shape immune activation thresholds, persistence, and fate decisions. Effective innate immune responses therefore depend not only on appropriate receptor and cytokine signals, but also on sufficient metabolic capacity to support those responses over time. Glucose, amino acids, and fatty acids can be channeled through glycolysis, the tricarboxylic acid (TCA) cycle, and oxidative phosphorylation (OXPHOS), or diverted toward lactate and biosynthetic pathways, providing distinct combinations of energy, redox balance, and intermediates for signaling and epigenetic regulation. By reprogramming enzyme expression and nutrient uptake, innate immune cells tune the contribution of these pathways to match functional demands, thereby establishing metabolic states that are more or less supportive of sustained immune function. To achieve this, innate immune cells reprogram gene and protein expression, altering metabolic enzyme activity and enhancing nutrient uptake and downstream pathways for energy production and biosynthesis to support specific cellular functions (17). This reprogramming involves upregulation of glycolytic flux, the pentose phosphate pathway (PPP), the TCA cycle, the mitochondrial respiratory chain, and fatty acid and amino acid metabolism (18).

In tumors, a metabolically licensed state can be context dependent, varying with cell type, tumor region, and disease stage. For example, macrophages can adopt diverse functional states with distinct metabolic features. M1-like states are often associated with increased glycolysis and expression of PFKFB3 and glucose transporters, whereas M2-like states can show greater utilization of glutamine (Gln) and fatty acids to support oxidative metabolism (17, 19). During tumor progression, spatial differences in oxygen availability, nutrient supply, and inflammatory signals can promote distinct metabolic programs in macrophages across different tumor regions (20). Furthermore, spatial transcriptomics analyses of lung cancer cohorts revealed that a specific TAM subset with lipid-associated and protumorigenic features expands with disease progression, demonstrating disease stage-dependent metabolic programs (21).

Despite this context dependence, metabolically licensed cells are generally characterized by sufficient nutrient uptake, preserved glycolytic reserve and spare respiratory capacity, maintained mitochondrial membrane potential, controlled redox balance, and the ability to sustain antitumor effector functions such as cytotoxicity, cytokine production, or antigen presentation. Metabolic adaptation alone does not constitute licensing; suppressive innate populations may retain substantial metabolic capacity when that capacity supports immunoregulatory rather than antitumor functions. The relative importance of these features, as well as their reversibility, may vary across innate immune populations and tumor contexts.

In contrast, metabolic restriction can arise from both direct limitations in metabolic capacity and active stress-responsive signaling that reprograms immune-cell function; these mechanisms frequently interact and reinforce one another within the TME. The term metabolic licensing used here is distinct from canonical NK-cell licensing or education, which refers to functional calibration through inhibitory receptor recognition of self-MHC class I.

In this section, we review how metabolic pathways license innate immune cells into functionally active states, focusing on glycolysis, mitochondrial respiration, and amino acid availability.

### 2.2 Glycolysis and acute activation

Among the metabolic programs that contribute to metabolic licensing, glycolysis is one of the earliest and most extensively studied pathways. In the activated state, immune cells reprogram signaling pathways such as mTORC1 and Akt, which enhance glucose uptake and effector cytokine secretion. Through such metabolic regulation, innate immune cells sustain their antitumor functions and maintain appropriate cell fate.

The principal pathways of glucose metabolism include glycolysis, the PPP, and the TCA cycle. Glycolysis converts glucose into pyruvate, which has two major fates: (1) entry into the TCA cycle and OXPHOS to generate large amounts of ATP, or (2) rapid conversion to lactate, generating less ATP but supporting high rates of biosynthesis and proliferation (22). Highly proliferative cells, including immune and tumor cells, often favor the latter route to rapidly generate energy and metabolic intermediates (22). Upon inflammatory stimulation, the transition from mitochondrial OXPHOS toward glycolysis-dependent metabolism is regulated by rate-limiting enzyme activities and is tightly linked to the execution of proinflammatory transcriptional programs (23, 24). The increased glycolytic flux also feeds the PPP and fatty acid synthesis pathways, providing intermediates required for nucleotide and lipid synthesis. Such reprogramming has been documented in antitumor innate immune cells, including NK cells, DCs, and macrophages (**Figure 1A**).

**Figure 1:**
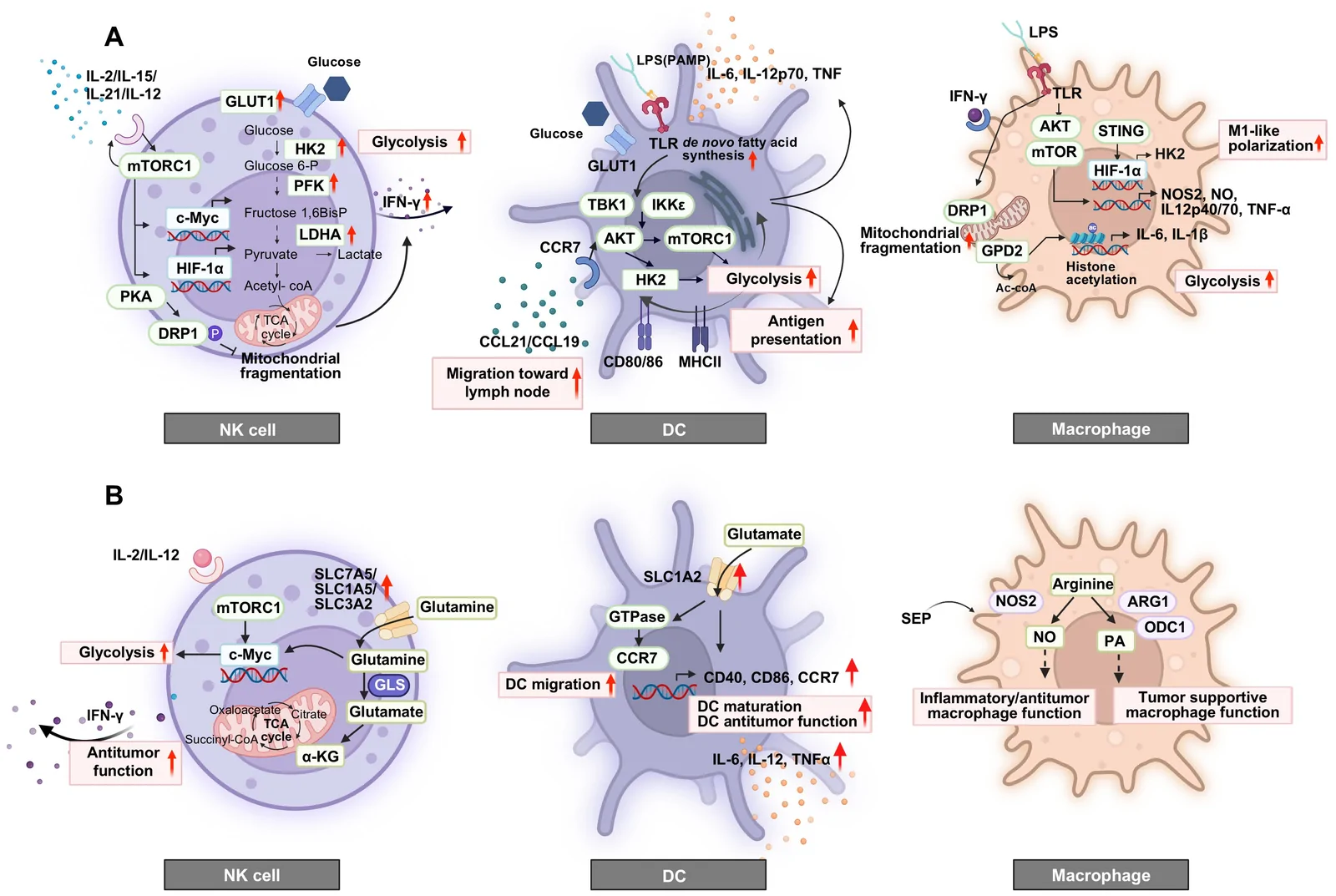
Immune cell activation and metabolic licensing. **(A)** Glycolysis and mitochondrial regulation in NK cells, dendritic cells, and macrophages. When NK cells are activated by IL-2, IL-15, IL-21, or IL-12, mTORC1 induces c-Myc and HIF-1α, promoting expression of GLUT1 and glycolytic enzymes (HK2, PFK, and LDHA); PKA-mediated DRP1 phosphorylation suppresses mitochondrial fragmentation and supports mitochondrial integrity. Upon PAMP sensing, TBK1/IKKϵ–AKT–mTOR signaling in dendritic cells increases glycolysis, which supports ER expansion, *de novo* fatty acid synthesis, cytokine production (IL-6, IL-12p70, and TNF), and upregulation of CD80/CD86 and MHC class II, as well as CCR7-dependent migration to lymph nodes. In macrophages, LPS and IFN-γ promote M1-like Inflammatory activation. GPD2-mediated glucose oxidation increases acetyl-CoA for histone acetylation and inflammatory gene expression, while TLR signaling activates STING/HIF-1α and PI3K/AKT/mTOR pathways, inducing HK2 expression and NOS2, NO, TNF-α and IL-12p40/70 production. DRP1-mediated mitochondrial fragmentation is also associated with inflammatory macrophage activation. **(B)** Amino acid metabolism in NK cells, dendritic cells and macrophages. Cytokine-activated NK cells upregulate amino acid transporters, including SLC7A5, SLC1A5, and SLC3A2, supporting mTORC1–c-Myc signaling, glycolysis, and NK-cell effector function. Activated DCs upregulate SLC1A2, which supports the expression of activation and costimulatory molecules (CD40 and CD86), cytokine production (IL-6, IL-12, and TNF-α), and migration through GTPase-CCR7 axis. In macrophages, distinct arginine metabolic pathways are associated with different functional states: NOS2-mediated NO production is associated with inflammatory/antitumor macrophage function, whereas ARG1- and ODC1-dependent polyamine synthesis is associated with tumor supportive macrophage function.

#### 2.2.1 NK cell activation

NK cells undergo metabolic reprogramming during maturation and development (25). In the steady state, NK cells depend largely on mitochondrial respiration, but activation induces glucose uptake and glycolytic flux, which support ATP production and cytotoxic molecule synthesis (26). Enhanced glycolysis supports persistence and effector function of NK cells in nutrient-deprived TMEs (26).

This is experimentally supported by cytokine-driven NK cell activation models. In NK cells activated with poly (I:C) *in vivo*, this glycolytic state is driven by mTORC1 activation, which promotes expression of glucose transporter 1 (GLUT1) and glycolysis-related enzymes such as, hexokinase 2 (HK2), and LDHA and is critical for IFN-γ production (27). mTORC1 also contributes to glycolytic control by inducing transcription factors hypoxia-inducible factor-1α (HIF-1α) and c-Myc, in murine NK cells stimulated with IL-2/IL-12 (25). Exposure to high concentrations of IL-15 activates mTOR, stimulating growth and nutrient uptake and sustaining NK cell proliferation and acquisition of cytolytic potential (25).

This is also supported by mouse tumor models. NK cell activated with IL-21 and IL-15 *in vivo* shows enhanced murine colorectal carcinoma, melanoma, and lymphoma tumor control with glycolysis upregulation in a LDHA-dependent manner (28). NK cell-specific deletion of LDHA demonstrates glycolysis is critical for NK cell-mediated tumor surveillance *in vivo* (29).

Glycolytic metabolism also contributes to NK cell education and supports effector function associated with signaling through killer immunoglobulin-like receptors (KIRs) (30). Educated NK cells, which express inhibitory KIRs that recognize self-HLA ligands, display greater glycolytic activity and mitochondrial-dependent glutaminolysis than uneducated NK cells (30).

Collectively, these studies show that cytokine-driven mTORC1-HIF-1α-c-Myc signaling establishes a glycolytic state that licenses NK cells for sustained cytotoxicity and IFN-γ production, particularly in nutrient-deprived tumor microenvironments.

#### 2.2.2 Dendritic cell activation

DCs play an essential role in the priming of T cell responses by antigen presentation (31). When DCs detect changes in the homeostatic state caused by pathogens or tissue-derived inflammatory signals, they shift from the resting state to the active state (32). In lipopolysaccharide (LPS)-mediated mouse DC activation model, the glycolytic flux is increased, which supports the *de novo* synthesis of fatty acids, with expansion of the endoplasmic reticulum (ER) and Golgi producing cytokines and activation markers (33). This rapid induction of glycolysis also supports activation and function of DCs, which is controlled by activation of rate-limiting glycolytic enzyme HK2 by the kinase TBK1, IKKϵ, Akt, and mTORC1 (33, 34). In line with this, the LPS-induced production of interleukin 6 (IL-6), IL-12p70, tumor-necrosis factor (TNF), and antigen presentation were impaired by 2-deoxyglucose (2-DG), supporting the importance of glycolysis in DC activation (33). Also, tumor-infiltrating DCs exhibited elevated glycolysis and STING-dependent antitumor functions in murine colon cancer cell (35). This is supported by increased glycolysis levels in DCs within NSCLC tissue, whereas LDHA deficiency impaired DC-mediated antitumor immunity and glycolytic activity in B16F10 melanoma model (35). It is also reported that early induction of glycolysis is important for cytoskeletal modification and DC migration to draining lymph nodes by oligomerization of CCR7 monomers, the receptor for the chemokines CCL21 and CCL19 (34).

Together, these studies indicate that rapid induction of glycolysis licenses multiple aspects of DC biology—activation, migration, cytokine production, and antigen presentation—by supplying both energy and biosynthetic precursors in a time-sensitive manner. DCs that cannot engage this glycolytic burst fail to fully transition from a resting state to a functionally competent antigen-presenting state despite exposure to activating signals.

#### 2.2.3 M1-like macrophage polarization

Metabolic pathways are associated with the fate of macrophages, which display distinct phenotypes and effector functions. M1-like macrophages are generated upon stimulation by inflammatory signals, such as Toll-like receptor (TLR) stimulation, ROS and IFN-γ (36). LPS activates TLR4-MyD88/TRIF-NF-κB/IRF signaling, whereas IFN-γ activates JAK–STAT1 signaling. Together, these pathways induce inflammatory genes such as Nitric oxide synthase 2 (NOS2), TNF-α, IL-6, and IL-1β and promote glycolytic reprogramming and antitumor functions (37).

However, LPS plus IFN-γ is primarily used as an experimental model of classical macrophage activation; within tumors, related inflammatory programs may instead be induced by endogenous TLR ligands, interferons, and other tumor- or immune-derived signals. For example, in murine colorectal cancer model, HMGB1 released by tumor cells induced M1-like macrophage polarization via the TLR4-MyD88-NFκB-NLRP3 pathway (38). In general, pro-inflammatory macrophages are known to display a high dependence on glycolysis, with impaired TCA cycle and mitochondrial oxidative phosphorylation, fatty acid biosynthesis (39).

This glycolysis dependence supports their inflammatory function (40, 41) by various mechanisms, including epigenetic modification, HIF-1α activation, and mTOR signaling. When activated by bacterial LPS, mitochondrial glycerol 3-phosphate dehydrogenase (GPD2) regulates glucose oxidation to promote inflammatory responses (42). GPD2, a component of the glycerol phosphate shuttle, can boost glucose oxidation to fuel the production of acetyl coenzyme A via Glycerol 3-phosphate shuttle (GPS) pathway, which facilitates acetylation of histones and induction of genes encoding inflammatory mediators, such as IL-6 and IL-1β (42). In addition, glycolysis in macrophage can promote STING-TBK1-IRF3 signaling cascade, which facilitates transcription of HIF-1α and further promote HK2-dependent glycolysis and inflammatory functions in liver fibrosis model (40). Because this evidence was obtained outside the tumor setting, it provides mechanistic support rather than direct evidence of antitumor metabolic licensing in the TME. In tumor model, STING activation has also been shown to induce IFN-β production and promote inflammatory, antitumor macrophage state (43). In another study using murine lung cancer model, PcrV-mediated TLR4 activation promoted M1-like polarization through PI3K/AKT/mTOR signaling, accompanied by increased glycolytic activity and increased NOS2 expression, NO production, IL-12p40/70, and TNF-α (44).

Together, these studies indicate that glycolytic reprogramming can support inflammatory macrophage functions through multiple mechanisms, including GPD2-dependent glucose oxidation and STING–HIF-1α- or PI3K/AKT/mTOR-associated signaling. However, the relationship between glycolysis and macrophage state is context dependent, and glycolytic activity alone does not define a fixed inflammatory or tumor-supportive phenotype.

### 2.3 Mitochondrial fitness and persistence

Beyond glycolysis, mitochondrial fitness is essential for sustaining antitumor activity and metabolically licensed innate immune responses in the TME. Mitochondria support immune function not only through OXPHOS and the generation of metabolic intermediates but also by integrating inflammatory signals and producing ROS and other signaling mediators. Their function is tightly regulated by dynamic structural processes, including fusion, fission, and selective degradation, which influence metabolic flexibility, persistence, and fate decisions in immune cells (23).

Mitochondrial fusion can stabilize membrane potential, preserves organelle morphology and function, and limit excessive inflammatory signaling (23). In contrast, mitochondrial fission can increase ROS production and promote mitophagy or apoptosis through cytochrome c release. In innate immune cells, mitochondrial fitness is therefore critical for sustaining immune responses and effector functions, and multiple studies have reported close correlations between mitochondrial state and immune cell function. In particular, mitochondrial fitness supports metabolic flexibility and persistence in antitumor innate immune cells such as NK cells and M1-like macrophages.

These apparently conflicting observations suggest that mitochondrial remodeling is context dependent. Rather than dictating macrophage polarization per se, mitochondrial dynamics may contribute to the metabolic flexibility required to support distinct macrophage programs under different environmental conditions.

#### 2.3.1 NK cells

In NK cells, mitochondrial fitness provides the metabolic reserve required to sustain antitumor activity after initial glycolytic licensing. Excessive mitochondrial fragmentation, ROS accumulation, and loss of membrane potential are associated with loss of NK-cell metabolic fitness and function, whereas preservation of mitochondrial integrity supports sustained effector activity (45, 46).

*Ex vivo* expansion studies have shown that activation increases both glycolysis and OXPHOS. Feeder-based expansion increases extracellular acidification rate (ECAR) and oxygen consumption rate (OCR), indicating enhanced glycolytic and mitochondrial respiratory capacity and supporting a link between metabolic fitness and NK cell effector function (47).

Mitochondria thus play a critical role in NK cell fate, and mitochondrial dysfunction impairs NK activity in cancer (48, 49). In hypoxic TME from orthotopic 4T1 tumor model, mTOR–DRP1 signaling was associated with excessive mitochondrial fission, limiting survival of tumor-infiltrating NK (TINK) cells through increased ROS and cytochrome c release (50); restoration of mitochondrial morphology enhances NK antitumor activity. Conversely, DRP1 phosphorylation via PKA signaling can suppress mitochondrial fragmentation and improve NK function under acidic stress in the TME, with enhanced antitumor efficacy in various human cancer cell models (51). PGC-1α-dependent mitochondrial biogenesis has also been shown to be crucial for optimal NK antitumor responses in murine melanoma cancer (52). Collectively, these studies highlight that mitochondrial fitness—encompassing intact respiratory capacity, balanced fusion–fission dynamics, and controlled ROS production—licenses NK cells for persistent antitumor function and survival in the TME.

#### 2.3.2 Macrophages

In macrophages, mitochondrial dynamics and respiratory activity are closely linked to functional state, but their effects are strongly context dependent. Changes in fusion, fission, membrane potential, and OXPHOS can accompany distinct inflammatory or tumor-supportive programs rather than defining a single polarization trajectory.

Mitochondrial dynamics are closely associated with macrophage functional states and polarization programs. The functional state of mitochondria is closely linked to regulation of metabolism in macrophages, with mitochondrial dysfuction priming macrophage toward glycolytic phenotype (53). Studies have shown that M1-like polarization is associated with LPS-dependent DRP1 dephosphorylation and mitochondria fragmentation, whereas M2-like polarization is associated with mitochondrial fusion and upregulated mitochondrial fusion mediators, MFN1/2 and FAM73A/B (53, 54). In murine breast cancer model, MFN1 silencing using shMFN1 inhibited mitochondrial fusion and shifted tumor-associated macrophages from an M2-like toward a more M1-like state, accompanied by enhanced antitumor activity (55). These changes were associated with delayed tumor growth and increased production of proinflammatory cytokines (55).

Optic atrophy 1 (OPA1), a mitochondrial-shaping protein that controls fusion, has emerged as a key regulator of macrophage metabolism. OPA1 dysfunction impairs M1-like polarization, leading to accumulation of TCA intermediates, impaired NF-κB signaling, and increased ROS generation. Loss of OPA1 can cause defective OXPHOS, reduced membrane potential, and a shift toward an M2-like bioenergetic state driven by glycolysis (56). In contrast, a preprint study suggests that OPA1 deficiency may promote NF-κB activation and inflammatory macrophage features under different experimental conditions (57). Despite these differences, available data converge on the view that mitochondrial function and fusion–fission balance critically regulate M1/M2-like macrophage polarization. Together, these findings indicate that mitochondrial dynamics contribute to macrophage metabolic and functional plasticity, although the direction of this effect varies with experimental and disease context.

#### 2.3.3 Dendritic cells

In dendritic cells, mitochondrial state is associated with redox metabolism and OXPHOS capacity, supporting their role in T cell priming and antitumor activity. During tumor progression, sustained mitochondrial potential and mitochondrial fusion licenses antitumor function of DCs. In contrast, reduced mitochondrial membrane potential and mitochondrial fragmentation can restrict these functions.

Intratumoral cDC1s exhibited discrete mitochondrial states, and OPA1-mediated mitochondrial metabolism promoted cDC1 antitumor responses in murine tumor model (58). DC-specific deletion of OPA1 in mice impaired mitochondrial fitness, resulting in defects in DC antigen uptake, migration to the tumor-draining lymph nodes, T cell priming, and CD8^+^ T cell effector function, along with increased growth and weight of tumors (58). Transcriptomic data demonstrated that OPA1-NRF1 axis supports OXPHOS activity and cDC1 function in priming CD8^+^ T cells (58).

### 2.4 Amino acid availability and cytokine-driven metabolic priming

Amino acid metabolism is important for supporting the functions of both tumor and immune cells (59). Amino acids provide essential nutrients for immune responses, and dysregulated amino acid availability is one mechanism that contributes to impaired antitumor immunity (60). Among these nutrients, glutamine is an important source of metabolic fitness in innate immune cells and influences both cell fate and immune function. A major mitochondrial fate of glutamine is replenishment of the TCA cycle through the glutamine–alpha-ketoglutarate (α-KG) axis (61). In this pathway, glutamine is converted to glutamate by glutaminase (GLS), and glutamate is subsequently converted to α-KG and enters the TCA cycle (61). The production of α-KG can therefore influence the metabolic programs and functions of innate immune cells, including macrophages and NK cells (**Figure 1B**).

In NK cells, amino acid transporters including SLC7A5, SLC1A5, and SLC3A2 are upregulated in response to IL-2 and IL-12 stimulation, and glutamine plays an important role in NK cell function by regulating mTORC1 signaling (60, 62). Glutamine uptake regulates the c-Myc dependent activation of NK cells (63). Another study also reported that the transcription factor c-Myc is essential for IL-2/IL-12-induced metabolic response and effector functions in NK cells, which are regulated by SLC7A5 and glutamine (64). In cytokine-activated NK cells, mTORC1 signaling activation and c-Myc expression are induced and through glutamine transport via SLC1A5, which increased glycolytic activation and production of IFN-γ (64). In line with this, glutamine deprivation in TME can be another source for NK cell dysfunction, with reduced of c-Myc protein expression, which is validated *in vitro* via glutaminolysis inhibitor, BPTES or DON (64).

In Dendritic cells, glutamine also plays a central role in DC activation and maturation. *In vivo* experiments demonstrated that SLC1A2 is upregulated in activated DCs to boost glutamate uptake, which promotes DC maturation, migration, cytokine secretion, and antitumor function (65). DC-specific SLC1A2 deletion impaired DC maturation, as evidenced by reduced expression of CD40, CD86, CCR7, accompanied by decreased production of the inflammatory cytokines IL-6, IL-12, and TNF-α (65). Furthermore, glutamate signaling induced Sema3A/small GTPase/CCR7 axis, which drove dynamic cytoskeletal remodeling and enhanced DC migration and interaction with T cells, in murine lung cancer and T lymphoma model (65).

In macrophages, distinct arginine metabolic pathways are associated with antitumor or tumor supportive functional states. Inflammatory/M1-like macrophage states can preferentially metabolize arginine via NOS2 to produce nitric oxide (NO), supporting antitumor activities, whereas tumor-supportive/M2-like macrophages can engage arginase 1 (ARG1) and ornithine decarboxylase 1 (ODC1)-dependent polyamine (PA) synthesis (66). Sepiapterin (SEP), the endogenous BH_4_ (NO synthase cofactor) precursor, redirects arginine metabolism from PA to NO synthesis and shifts macrophages from M2-like toward more inflammatory M1-like features (66). Consistent with this shift, SEP-treated macrophages showed increased antigen-presenting and antitumor activity. In the MMTV-neu mouse model, SEP treatment increased the proportion of M1-like TAMs and was accompanied by reduced tumor growth (66).

These studies show that amino acid availability can support metabolic licensing of antitumor innate immune responses, although the functional outcome depends on how individual amino acids are metabolized. In NK cells, cytokine-induced amino acid transport and c-Myc-dependent reprogramming support cytotoxic function under metabolic stress. In macrophages, diversion of arginine metabolism toward NO production can support antitumor activity, whereas ARG1-dependent polyamine synthesis is associated with tumor-supportive states. In DCs, glutamine transport similarly supports antitumor function.

### 2.5 Acute versus sustained metabolic fitness in innate immunity

Under steady state, immune cells exhibit low biosynthetic activity and dependence on OXPHOS and Fatty acid oxidation (FAO) to meet their basal energy requirements (67). Upon activation, they rapidly induce metabolic reprogramming to meet heightened energy needs, by enhancing glycolysis, the TCA cycle and glutaminolysis (67). When these metabolic changes are sustained, the accumulation of metabolic intermediates such as acetyl-CoA, fumarate, succinate, nicotinamide adenine dinucleotide (NAD+) and mevalonate, plays a critical role in establishing sustained metabolic fitness in immune cells (67). Furthermore, this metabolic intermediate can control the methylation (H3K4me3) and acetylation (H3K27ac) of histones, thereby inducing epigenetic reprogramming (68). This epigenetic reprogramming can result in sustained alteration of metabolic activity, with immune cells showing memory-like phenotypes.

During immune activation, glycolysis is significantly upregulated in antitumor immune cells, such as NK cells, DCs, and M1-like macrophages. Generally, this is driven by activation of Akt/mTOR/HIF-1α pathway and expression of glycolysis-mediating enzymes, including glucose transporter and rate-limiting enzymes (67). Although it is reported that activated immune cells metabolically shift from OXPHOS to glycolysis, mitochondrial function and fitness are also important for their persistence.

In addition, ROS formation from mitochondria respiration is important for establishing the memory phenotype, including increased cytokine production, mTOR phosphorylation, HIF-1α stabilization, and lactate production (67, 69). For example, changes in the cellular redox state can regulate physiological function and fate of macrophages by transcription regulation, such as HIF-1α (69). Supporting this, monocytes activated by oxidized low-density lipoprotein (oxLDL) induced proinflammatory macrophage polarization, which was inhibited through inhibition of HIF-1α or mTOR, or antioxidant (69). Therefore, mTOR dependent ROS production controls metabolic reprogramming in immune cells with stabilization of HIF-1α (69). In melanoma model, reuterin, a gut microbiota-derived metabolite, shifted macrophages from an M2-like toward a more inflammatory state through the AHR-ROS-HIF-1α axis, accompanied by reduced tumor growth (70).

Antitumor innate immune cells can also use glutamine to replenish the TCA cycle through α-KG production. In monocytes, glutamine-dependent accumulation of fumarate has been linked to persistent metabolic and epigenetic reprogramming (71). Following β-glucan stimulation, monocytes increase glycolysis, glutamine metabolism, and cholesterol metabolism, while fumarate accumulation promotes H3K4me3 at promoters of inflammatory cytokine genes through inhibition of KDM5 histone demethylases and contributes to HIF-1α stabilization (71). These trained-immunity studies were performed primarily in non-tumor settings and therefore provide mechanistic evidence for metabolite-dependent epigenetic remodeling rather than direct evidence of antitumor metabolic licensing within the TME.

Together, these observations suggest that metabolic licensing is not limited to the initial activation of innate immune cells but can be reinforced by mitochondrial function and metabolite-driven epigenetic remodeling. These adaptations sustain antitumor competence over time and determine whether innate immune cells remain metabolically licensed or become susceptible to metabolic restriction within the tumor microenvironment.

## 3 Metabolic restriction and dysfunction in the TME

### 3.1 Glucose deprivation and glycolytic restriction

Tumor cells frequently maintain high glycolytic activity even under normoxic conditions, a phenomenon known as the Warburg effect. By maintaining high glycolytic flux even when oxygen is available, tumor cells generate metabolic intermediates that support proliferation (72). Their high glucose consumption creates a nutrient-scarce tumor microenvironment (72). Because immune cells also require glucose for activation and effector function, competition with tumor cells imposes metabolic stress on antitumor immune populations.

High glucose consumption by cancer cells is supported by increased glucose uptake and expression of glycolytic enzymes (73). Oncogenic and stress-responsive pathways, including PI3K–AKT, HIF-1α, Ras, mTOR, c-Myc, and p53, regulate glucose uptake and glycolytic activity (74). Under hypoxic condition in human melanoma cancer cells, HIF-1α induces the expression of GLUT1, lactate dehydrogenase (LDH), and HK2 (75). HIF signaling also cooperates with oncogenic MYC, which upregulates multiple glycolytic enzymes (76). c-Myc has been linked to increased expression of L-type pyruvate kinase, GLUT1, GLUT2, LDHA, and phosphofructokinase (77, 78).

Within the TME, antitumor innate immune cells must compete with tumor cells for the glucose required to sustain glycolysis. NK cells isolated from ovarian tumors show impaired tumor-cell killing together with reduced glycolysis and OXPHOS (79). By contrast, NK cells engineered to improve glycolytic capacity and metabolic adaptation are more resistant to tumor-associated suppression and display enhanced antitumor activity (79). These findings indicate that nutrient deprivation can compromise NK cell metabolic fitness and effector function (79).

Across multiple solid tumors, high tumor glycolytic signatures are associated with reduced immune infiltration and poorer clinical outcomes (80). NK cell-intrinsic mechanisms can further reinforce glycolytic restriction. Fructose-1,6-bisphosphatase 1 (FBP1), a rate-limiting enzyme in gluconeogenesis that opposes glycolysis, is upregulated in tumor-infiltrating NK cells during murine lung cancer progression (81). Increased FBP1 expression is associated with reduced NK cell glycolysis, impaired antitumor function, and advanced tumor stage (81).

Collectively, these findings identify glucose availability as an important licensing factor for NK cell activation and antitumor function. When tumor cells dominate glucose consumption, innate immune cells experience glycolytic restriction that weakens metabolic fitness and effector activity (**Figure 2A**).

**Figure 2:**
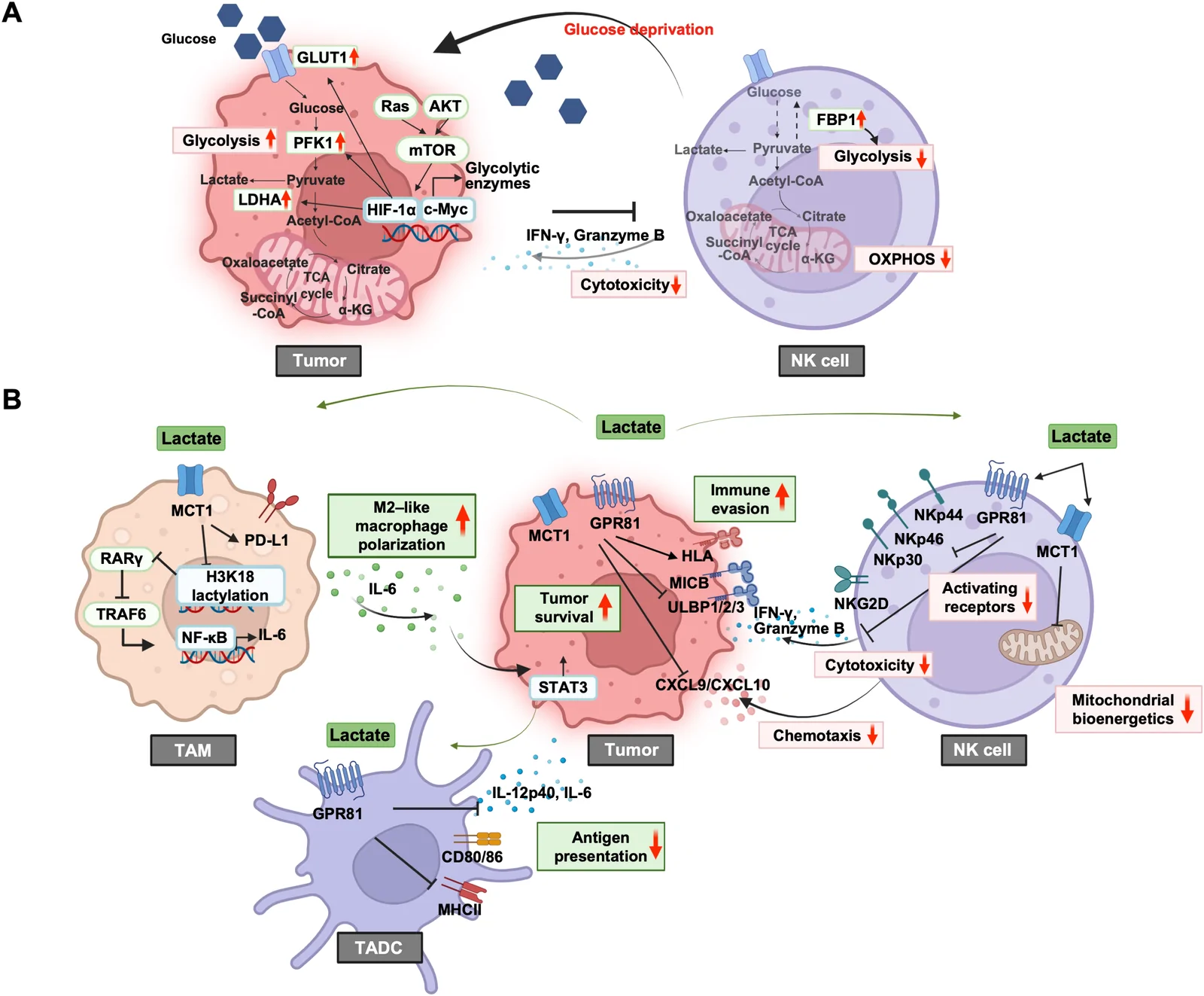
Immune cell dysfunction and metabolic restriction under glucose deprivation and lactate accumulation. **(A)** Glucose competition. Oncogenic Ras and PI3K–AKT–mTOR pathways promote HIF-1α- and c-Myc-dependent expression of glucose transporters and glycolytic enzymes, including GLUT1, PFK1, and LDHA, thereby increasing glycolytic flux in tumor cells. The resulting glucose competition within the TME limits glycolysis, OXPHOS, cytotoxicity, and IFN-γ and granzyme B production in NK cells. Increased FBP1 expression further suppresses NK cell glycolysis and contributes to dysfunction. **(B)** Lactate accumulation. Lactate exposure is associated with impaired NK-cell mitochondrial bioenergetics, reduced expression of activating receptors such as NKp30, NKp44, and NKp46, and decreased cytotoxicity and reduced IFN-γ and granzyme B production; GPR81 signaling and MCT1-dependent lactate transport have been implicated in these suppressive effects. In tumor cells, GPR81 signaling reduces the expression of NKG2D ligands, including MICB, ULBP1, ULBP2, and ULBP3, while increasing inhibitory HLA expression, thereby weakening NK cell recognition. Reduced CXCL9 and CXCL10 production can further limit NK cell chemotaxis. Lactate also promotes M2-like macrophage polarization through H3K18 lactylation and RARγ–TRAF6–NF-κB–IL-6 signaling, with subsequent STAT3 activation in tumor cells. In dendritic cells, lactate promotes tumor-associated phenotypes characterized by reduced MHC class II and costimulatory molecule expression, impaired antigen presentation, and decreased IL-6 and IL-12p40 production.

### 3.2 Lactate accumulation and acid stress

Tumor cells frequently increase glucose uptake and glycolysis, resulting in substantial lactate accumulation within the TME and suppression of immune-cell function. High intratumoral lactate levels correlate with advanced breast cancer grade, poor clinical outcome, and immune dysfunction (82, 83).

Lactate is exported from highly glycolytic cells through monocarboxylate transporters (MCTs) together with protons, contributing to extracellular acidification in the TME (84). However, lactate accumulation and low extracellular pH are mechanistically distinct. In colorectal liver metastasis patients, elevated lactate was associated with mitochondrial stress, ROS accumulation and apoptosis in tumor-infiltrating NK cells (85). Where low extracellular pH was examined experimentally, acidic condition independently impaired NK cell mitochondrial function and increased mitochondrial ROS (85).

Lactate can also act through receptor-mediated signaling, including GPR81, or enter cells through MCT-dependent transport. These mechanisms are distinct from the effects of extracellular acidosis. Lactate can impair NK-cell antitumor activity, alter gene expression through histone lactylation, and promote immunosuppressive cell states, thereby reinforcing immune suppression within tumors.

Several studies have linked lactate accumulation to NK cell dysfunction. In immunocompetent mouse models, tumors with reduced lactic acid production grew more slowly and showed increased NK cell infiltration, consistent with the inhibitory effect of lactic acid on NK cell cytokine production (82). In breast cancer, elevated lactate metabolism inversely correlates with NK cell activation signatures and is associated with unfavorable survival outcomes (86). Lactate exposure was associated with reduced expression of NK cell activating receptors, including NKp30, NKp44, and NKp46, and decreased IFN-γ and granzyme B production. GPR81 signaling and MCT-dependent lactate transport have been implicated in these suppressive effects (86). These changes are accompanied by impaired mitochondrial bioenergetics and lipid accumulation in NK cells and can be partially reversed by blocking lactate transport (86). In tumor cells, GPR81 signaling also decreases the expression of NK cell activating ligands, including MICB, ULBP1, ULBP2, and ULBP3 while increasing inhibitory HLA molecules, thereby weakening NK cell-mediated immune surveillance (86). Reduced CXCL9 and CXCL10 production further limits NK cell chemotaxis and cytotoxicity (86). Together, these effects suppress NK cell activation, migration, cytokine production, and tumor-cell killing.

Beyond its direct effects on NK cells, lactate can reshape innate immune-cell fate through histone lactylation. In gastrointestinal cancers, increased lactylation is associated with M2-like macrophage polarization, elevated PD-L1 expression, and reduced infiltration of cytotoxic immune cells (87). Tumor-derived lactate promotes H3K18 lactylation in macrophages and suppresses RARγ transcription (88). Reduced RARγ expression favors tumor-promoting macrophage polarization and enhances TRAF6-NF-κB-dependent IL-6 production, which subsequently activates oncogenic STAT3 signaling in colorectal cancer cells (88). Consistent with this mechanism, inhibition of lactate production in colorectal cancer cells with 2-DG increases macrophage RARγ expression, reduces H3K18 lactylation, and improves tumor control (88). These findings suggest that lactate-dependent epigenetic regulation contributes to the establishment of suppressive macrophage states.

Lactate also supports the accumulation and function of other immunosuppressive myeloid populations. Tumor-derived lactate can induce M2-like macrophage polarization and promote TNFSF9 expression through MCT1-dependent H3K18 lactylation (89). High tumor LDHA expression is associated with M2-like macrophage enrichment and disease progression in TCGA cohorts (90). Lactate-stimulated macrophages also produce IL-6 and promote epithelial–mesenchymal transition, angiogenesis, and tumor progression (90).

Lactate can also impair dendritic-cell activation and maturation. In multicellular tumor spheroid models, infiltrating monocytes differentiated into tumor-associated dendritic-like cells in response to tumor-derived lactic acid, with reduced CD1a, CD83, HLA-DR, and IL-12 expression (91). Supporting this, inhibition of lactate transporter MCT1 rescued their inhibitory phenotype, with enhanced IFN-β and IL-12 (92). Tumor-derived lactate can prevent presentation of tumor-specific antigens to other immune cells through GPR81 signaling (93). In a mammary tumor model, GPR81 signaling reduced MHC class II and costimulatory molecule expression in DCs and was associated with decreased IL-6 and IL-12p40 production and impaired T-cell activation (93).

Together, these studies identify lactate accumulation as an important mediator of metabolic restriction in the TME through receptor-mediated signaling, MCT-dependent transport, and epigenetic regulation. Extracellular acidosis represents a related but distinct stress that can independently impair innate immune function. In NK cells, lactate signaling and transport impair mitochondrial bioenergetics and effector function, whereas in macrophages and dendritic cells, lactate sensing and histone lactylation can favor tumor-supportive or dysfunctional states (**Figure 2B**).

### 3.3 Hypoxia, mitochondrial stress, and ROS

#### 3.3.1 Hypoxia

Rapid tumor growth frequently outpaces vascular development, resulting in regions of chronic hypoxia within solid tumors (94). Hypoxia stabilizes HIF-1α and alters metabolic adaptation within the tumor microenvironment. Hypoxic conditions can also perturb mitochondrial function and redox homeostasis, although mitochondrial dysfunction and ROS accumulation represent related but distinct stress responses rather than obligatory downstream consequences of hypoxia. In parallel, acidic conditions can independently disrupt mitochondrial integrity, leading to mitochondrial fragmentation, loss of membrane potential, and impaired bioenergetic function (51).

Beyond promoting tumor metabolic adaptation, hypoxia contributes to the establishment of an immunosuppressive microenvironment. HIF-1α induces the expression of chemokine ligand 26 (CCL26), which recruits CX3CR1-positive MDSCs into hypoxic tumor regions (95). Hypoxia also increases the expression of the ectonucleotidases CD39 and CD73, resulting in extracellular adenosine accumulation (96, 97). Adenosine subsequently binds to the A2A receptor (A2AR) on NK cells and suppresses their metabolic activity and cytotoxic function through inhibitory signaling pathways (96, 98). In addition to its effects on immune cells, HIF-1α dependent signaling also supports tumor adaptation through multiple oncogenic pathways under hypoxic conditions.

Within this metabolically restricted environment, innate immune cells can progressively lose effector functions. NK cells are particularly sensitive to hypoxia. NK cells cultured under hypoxic conditions *in vitro* show reduced expression of activating receptors, including NKp30, NKp44, NKp46, and NKG2D, together with diminished cytokine production and cytotoxicity (99, 100). HIF-1α-dependent induction of CD73 promotes adenosine production, while HIF-1α has also been linked to ADAM-mediated shedding of MICA from tumor cells, thereby reducing NKG2D-mediated tumor recognition (101, 102).

#### 3.3.2 Mitochondrial stress

Mitochondrial dysfunction represents a distinct component of metabolic stress in the TME and can be promoted by hypoxia as well as other tumor-associated conditions. In NK cells, hypoxic exposure has been associated with mitochondrial fragmentation, reduced mitochondrial membrane potential, increased mitochondrial ROS (mtROS), downregulation of OXPHOS-related genes, and impaired function (50, 103). Consistent with this, both NK cells cultured under hypoxic conditions and tumor-infiltrating NK cells displayed fragmented mitochondria together with increased expression of the mitochondrial fission regulator DRP1 (50). Dendritic cells also exhibit altered mitochondrial fitness within tumors. During tumor progression, OPA1–NRF1 signaling declined in intratumoral cDC1s, accompanied by reduced mitochondrial membrane potential and impaired cDC1 antitumor function (58).

#### 3.3.3 ROS

Reactive oxygen species (ROS) can arise from multiple sources within the TME and function as signaling mediators or drivers of oxidative stress depending on their magnitude and duration (104). Hypoxia can alter the balance between ROS production and clearance by affecting electron transport chain activity and antioxidant defenses (104). Hydrogen peroxide concentrations are elevated in tumor interstitial fluid isolated from murine breast cancers, and oxidative stress impaired NK-cell cytotoxic activity *in vitro*. IL-15 priming preserved NK-cell function under oxidative stress in association with increased expression of the antioxidant enzyme PRDX1 (105).

In tumor-associated dendritic cells, persistent oxidative stress can promote ER stress and XBP1 activation, leading to intracellular lipid accumulation, impaired antigen presentation, and defective priming of antitumor immune responses (106). ROS-driven lipid peroxidation generates reactive aldehydes such as 4-hydroxy-*trans* - 2-nonenal (4-HNE), which can induce protein-folding and ER stress. In BMDCs exposed to tumor-conditioned medium, XBP1 activation was associated with triglyceride accumulation and reduced capacity to stimulate T-cell proliferation, whereas DC-specific XBP1 deletion restored T-cell priming and antitumor function (106).

Collectively, hypoxia, mitochondrial dysfunction, and oxidative stress represent distinct but interacting components of metabolic restriction in the TME, with HIF-1α-dependent signaling mediating a subset of hypoxia-associated responses. Their relative contributions vary with cell type and local tumor conditions, but sustained activation of these stress pathways can impair mitochondrial fitness, antigen presentation, cytotoxicity, and other innate immune effector functions (**Figure 3A**).

**Figure 3:**
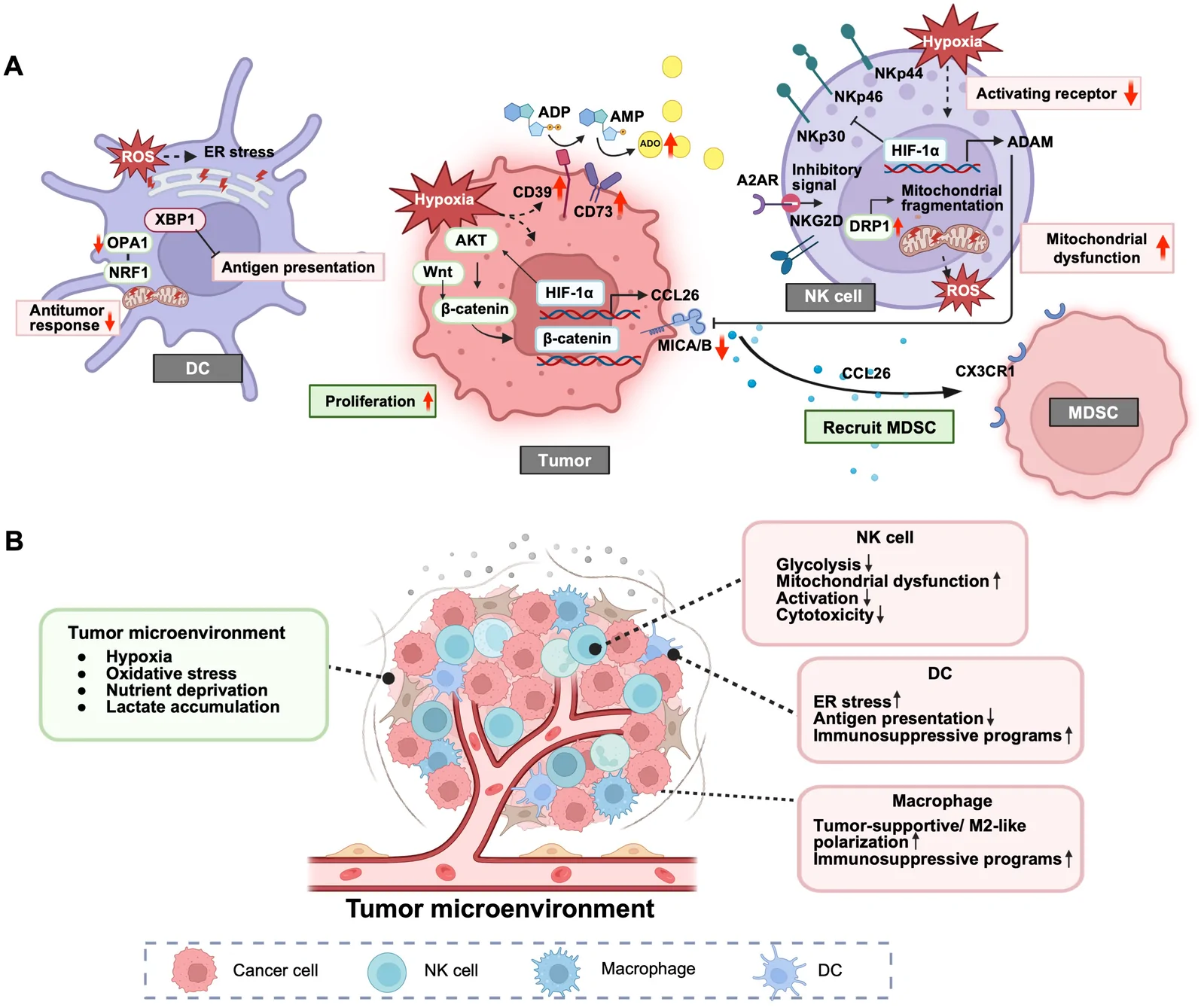
Hypoxia-associated metabolic restriction and innate immune dysfunction. **(A)** Hypoxia and oxidative stress. Hypoxia stabilizes HIF-1α and induces CCL26 expression, promoting the recruitment of CX3CR1^+^ MDSCs. Hypoxia increases CD39 and CD73 activity, leading to adenosine accumulation and A2AR-mediated inhibition of NK cells. Hypoxia is associated with reduced expression of NK cells activating receptors and can promote ADAM-mediated shedding of NKG2D ligands from tumor cells. Hypoxic stress is also associated with DRP1-dependent mitochondrial fragmentation in NK cells. In dendritic cells, oxidative stress and lipid peroxidation promote ER stress and XBP1 activation, impairing antigen presentation. Reduced OPA1–NRF1 signaling is associated with impaired mitochondrial fitness and diminished antitumor function in intratumoral cDC1s. HIF-1α-dependent Wnt, AKT, and β-catenin signaling further supports tumor-cell proliferation. **(B)** Integrated effects of metabolic restriction. Hypoxia, nutrient deprivation, lactate accumulation, and oxidative stress collectively impair innate immune function in the TME. Together, these stresses can impair glycolysis, mitochondrial fitness, activation, and cytotoxicity in NK cells, induce ER stress and defective antigen presentation in dendritic cells, and favor M2-like, tumor-supportive macrophage states and immunosuppressive functions.

### 3.4 Shared dysfunctional phenotypes of innate immune cells

Within the TME, persistent nutrient deprivation, hypoxia, and the accumulation of immunosuppressive metabolites progressively impair the metabolic fitness of innate immune cells. Although glucose deprivation, lactate accumulation, acidosis, hypoxia, and oxidative stress act through distinct mechanisms, their effects can converge at the level of innate immune function. Depending on cell type and local context, these stresses can impair glycolytic or mitochondrial fitness, cytokine production, antigen presentation, and cytotoxicity, contributing to dysfunctional or tolerant states (**Figure 3B**).

Tumor-infiltrating NK cells progressively acquire dysfunctional phenotypes characterized by reduced glycolytic capacity, mitochondrial fragmentation, impaired cytotoxicity, diminished cytokine production, and increased expression of inhibitory receptors such as PD-1, TIGIT, and CD39 (50, 81, 99, 107, 108). Together, these changes limit NK cell-mediated tumor control despite continued exposure to activating signals.

Dendritic cells can also acquire dysfunctional states in the TME. Tumor-derived lactate can impair antigen presentation and promote an immunosuppressive DC phenotype through mechanisms including GPR81 signaling (91, 93). These changes are associated with reduced MHC class II and costimulatory molecule expression, decreased IL-6 and IL-12 production, and impaired capacity to prime antitumor T-cell responses (93). As a result, dendritic cells exhibit reduced antigen presentation, impaired cytokine production, and diminished capacity to prime antitumor T cell responses, representing a metabolically restricted state.

Collectively, these observations indicate that metabolic restriction is not defined by a single metabolic abnormality but can emerge from the combined effects of persistent nutrient limitation, mitochondrial dysfunction, oxidative stress, and suppressive metabolite signaling. Although the resulting phenotypes vary across innate immune populations, they commonly include loss of metabolic fitness and one or more functional defects, such as impaired cytotoxicity, cytokine production, antigen presentation, or cellular persistence. At least some of these states remain reversible in experimental settings. Restoration of glycolytic capacity in NK cells, inhibition of tumor lactate production, or IL-15 priming under oxidative stress can partially restore innate immune function in specific models (79, 88, 105).

## 4 Metabolic programming of suppressive innate populations

### 4.1 Tumor-associated macrophages and monocytes

During early tumor development, inflammatory macrophages can infiltrate the TME and contribute to antitumor immunity by killing tumor cells and supporting adaptive immune responses. As tumors progress, however, chronic exposure to hypoxia, lactate, and other metabolic stresses favors the accumulation of M2-like or tumor-supportive TAM states through coordinated changes in glucose, lipid, and amino acid metabolism (109). In glucose-limited tumors, these macrophages can rely more heavily on OXPHOS and FAO, which support their survival and tumor-promoting functions (110, 111).

Lipid uptake is an important component of TAM metabolic adaptation. Increased CD36-mediated lipid uptake promotes fatty acid storage and supports mitochondrial FAO and OXPHOS (6). Cholesterol efflux can also enhance IL-4 signaling and induce transcriptional programs associated with tumor-supportive macrophage function (112).

Arginine metabolism is differentially regulated across macrophage states. NOS2-mediated nitric oxide production is associated with inflammatory macrophage programs, whereas ARG1-dependent conversion of L-arginine to urea and ornithine is associated with tumor-supportive macrophage states and can reduce local arginine availability (65, 113).

Collectively, increased lipid uptake and FAO, together with ARG1-dependent arginine metabolism, support the survival and immunosuppressive activity of TAMs in nutrient-limited tumors. These adaptations enable TAMs to deplete essential nutrients, produce tumor-supportive mediators, and remodel the TME in ways that favor tumor progression (**Figure 4A**).

**Figure 4:**
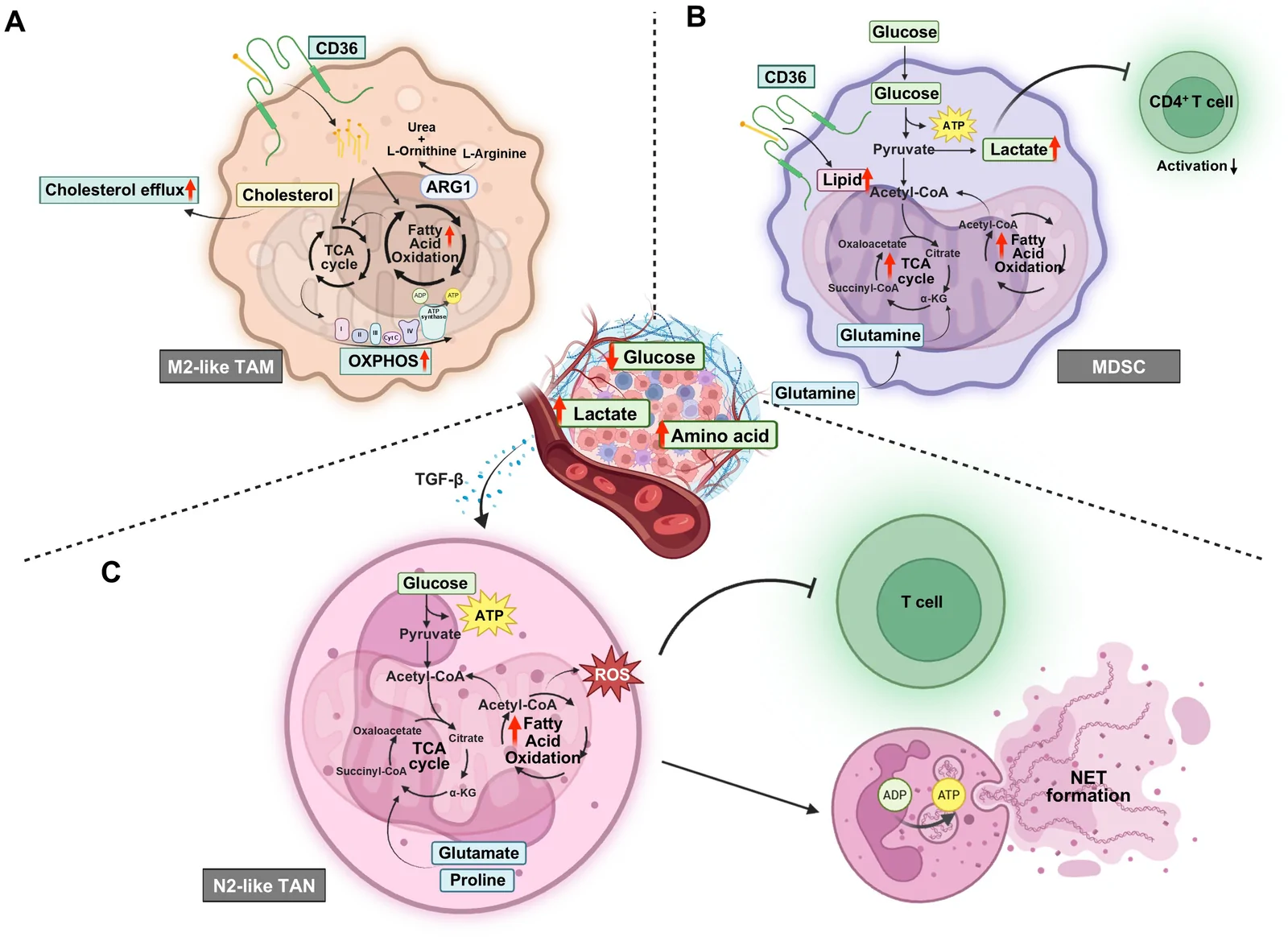
Metabolic adaptation of suppressive innate immune populations in the tumor microenvironment. **(A)** Tumor-associated macrophages. M2-like TAM states can exhibit increased CD36-mediated lipid uptake, FAO, and OXPHOS, while enhanced cholesterol efflux can support IL-4-associated tumor-supportive macrophage. Tumor supportive states can also express ARG1, which converts L-arginine into urea and L-ornithine and contributes to the suppression of T cell activation and tumor-supportive activity. **(B)** Myeloid-derived suppressor cells. Myeloid-derived suppressor cells (MDSCs) increase glycolysis, lactate production, CD36-dependent lipid uptake, FAO, and mitochondrial respiration. These metabolic programs provide flexibility and support their survival and immunosuppressive function in the TME. **(C)** Tumor-associated neutrophils. Tumor-associated neutrophils (TANs) display considerable metabolic plasticity within the TME. TGF-β and other tumor-derived signals promote the acquisition of N2-like, tumor-supportive phenotypes. Under glucose-limited conditions, these cells use glutamate and proline as alternative substrates for the TCA cycle and increase their reliance on FAO. These adaptations support ATP and ROS production, NET formation, and tumor-promoting activity.

### 4.2 Myeloid-derived suppressor cells

MDSCs arise from myeloid progenitors and accumulate in tumors, where they suppress T cell responses and contribute to an immunosuppressive TME (113). MDSCs can increase aerobic glycolysis and lactate production, which support their survival and suppressive activity and may further limit CD4^+^ T cell activation (114, 115). They also increase CD36-mediated lipid uptake and FAO, metabolic programs that help sustain immunosuppressive function (116) Consistent with this, pharmacological inhibition of FAO reduces their ability to suppress T cell proliferation and IFN-γ production (117).

Amino acid metabolism further contributes to MDSC fitness. L-glutamine replenishes the TCA cycle and supports MDSC viability and suppressive capacity under nutrient-limited conditions. Together, the ability to engage glycolysis, lipid metabolism, and glutamine-dependent TCA-cycle anaplerosis provides MDSCs with the metabolic flexibility required to persist and suppress antitumor immunity in environments that are unfavorable for conventional effector immune cells (118) (**Figure 4B**).

### 4.3 Neutrophils and tumor-associated neutrophils

Neutrophils develop in the bone marrow and are recruited to tumors by chemokines and growth factors such as CXCL1, CXCL2, CXCL6, IL-8, and G-CSF, where they can acquire tumor-associated phenotypes (119). TANs are heterogeneous and can display both antitumor and tumor-supportive features. N1-like and N2-like terminology has historically been used to describe these functional tendencies, with tumor-derived signals such as TGF-β promoting N2-like, tumor-supportive features in experimental models (119, 120).

To survive and function in hypoxic and nutrient-poor tumors TANs undergo substantial metabolic adaptation. Under glucose-limited conditions, they can maintain glycolytic activity while increasing their reliance on mitochondrial FAO, which supports ROS production and T cell suppression (121). When glucose is scarce, TANs can also use amino acids such as glutamate and proline to replenish the TCA cycle and sustain tumor-promoting functions, including neutrophil extracellular trap (NET) formation and metastatic spread (122). In breast cancer, these adaptations have been linked to liver metastasis. Tumor-derived glutamate may further promote the transition from tumor-killing N1-like states toward immunosuppressive N2-like phenotypes (123).

In summary, TANs adapt to hypoxic and nutrient-poor conditions by reprogramming glucose, lipid, and amino acid metabolism. These adaptations support ROS production, NET formation, metastasis, and suppression of antitumor immunity (**Figure 4C**).

### 4.4 Metabolic integration of suppressive innate programs

Although TAMs, MDSCs, and TANs arise from distinct developmental lineages, their adaptation to the TME involves several overlapping metabolic features. Chronic exposure to glucose deprivation, hypoxia, lactate accumulation, and oxidative stress can favor metabolic programs that support the survival, persistence, and immunoregulatory activity of suppressive innate populations.

These adaptations are not uniform across cell types. Increased lipid uptake, FAO, and OXPHOS contribute to the persistence and suppressive activity of M2-like TAMs and lipid-adapted MDSCs, whereas lactate-rich and hypoxic conditions can promote signaling and epigenetic programs associated with tumor-supportive macrophage and neutrophil states. Amino acid metabolism also contributes to cell type-specific ways, including ARG1-dependent arginine metabolism in TAMs, glutamine utilization in MDSCs, and glutamate and proline utilization in TANs.

Together, these observations illustrate how distinct innate immune populations can use different metabolic adaptations to maintain tumor-supportive functions under chronic metabolic stress. Importantly, the same metabolic pathways do not uniformly define suppressive states across cell types; their functional consequences depend on cellular identity and the local tumor context. This context dependence provides a rationale for therapeutic strategies that target suppressive metabolic programs while preserving antitumor innate immune function.

## 5 Shared metabolic sensors linking stress to innate immune fate

Innate immune cells in tumors are continuously exposed to changes in oxygen, nutrient availability, metabolites, and danger signals. These inputs are not sensed independently but are integrated through shared stress-responsive pathways that connect environmental conditions to metabolic reprogramming and cell fate. Hypoxia and nutrient-sensing pathways involving HIF-1α, mTOR, and AMPK, oxidative and organelle stress responses involving ROS, ER stress, and the unfolded protein response (UPR), and innate inflammatory pathways such as TLRs, inflammasomes, and cGAS–STING collectively shape whether innate immune cells maintain antitumor activity or acquire dysfunctional or suppressive states (**Figure 5A**).

**Figure 5:**
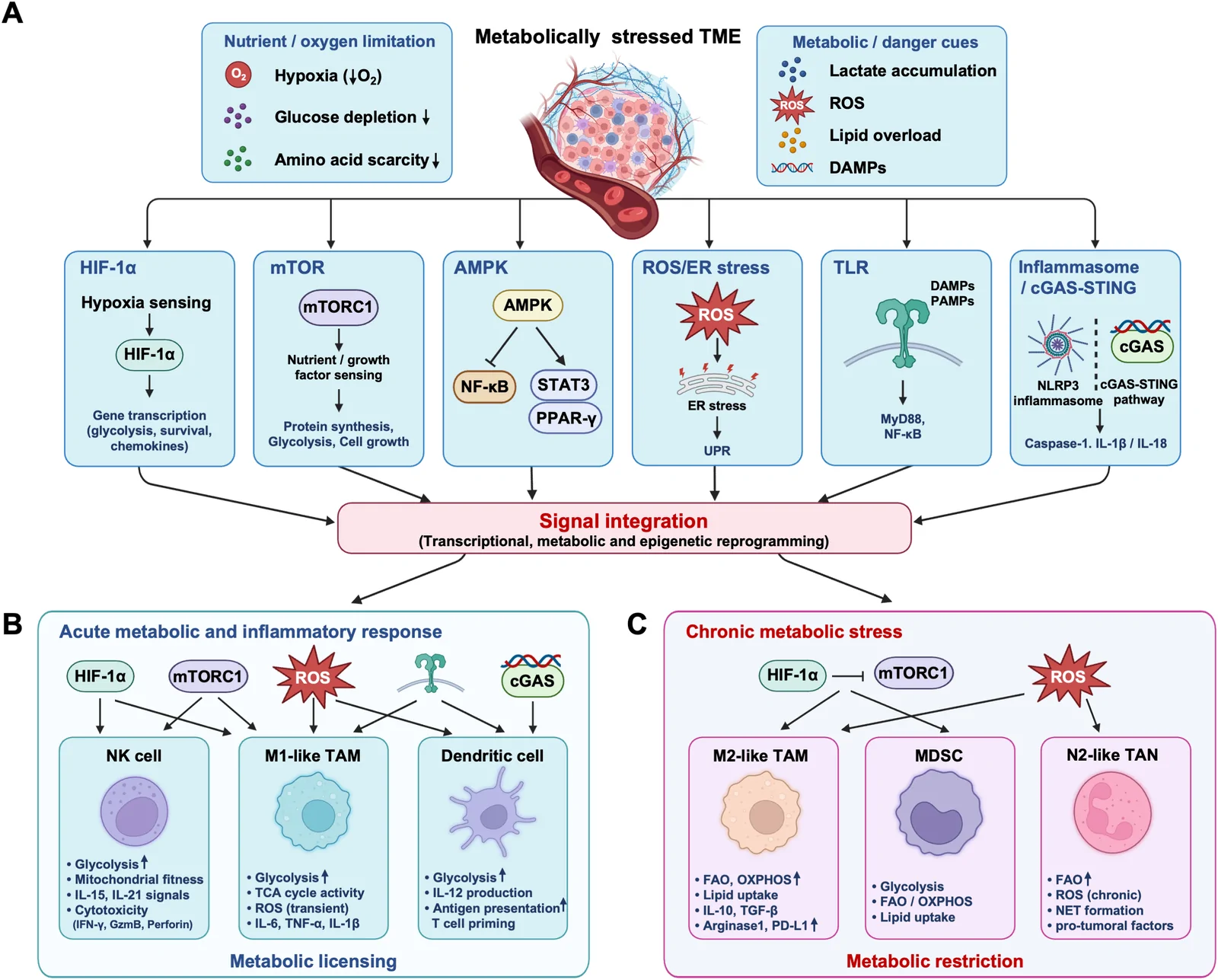
Stress sensing shapes innate immune cell fate. **(A)** Innate immune cells in the TME encounter oxygen, glucose, and amino acid limitation together with lactate accumulation, lipid overload, ROS, and diverse danger signals. These conditions engage HIF-1α-mediated hypoxia sensing, mTORC1- and AMPK-dependent nutrient and energy sensing, ROS and ER stress responses, and innate inflammatory pathways involving TLRs, NLRP3 inflammasomes, and cGAS–STING. Their interaction contributes to transcriptional, metabolic, and epigenetic reprogramming. **(B)** Metabolic licensing supports antitumor innate immune states. In NK cells, glycolysis, mitochondrial fitness, and IL-15 and IL-21 signaling support cytotoxic mediators, including IFN-γ, granzyme B, and perforin. In dendritic cells, glycolysis, IL-12 production, antigen presentation, and T cell priming support immune activation. In M1-like macrophages, glycolysis, TCA-cycle remodeling, transient ROS production, and inflammatory cytokines, including IL-6, TNF-α, and IL-1β, sustain inflammatory effector programs. **(C)** Metabolic restriction favors suppressive innate immune states. M2-like TAM states can exhibit increased FAO, OXPHOS, and lipid uptake together with expression of IL-10, TGF-β, ARG1, and PD-L1. MDSCs can engage glycolysis, FAO, OXPHOS, and lipid uptake to support suppressive activity. N2-like TAN states can exhibit increased FAO, sustained ROS production, NET formation, and other tumor-promoting features. Acute and chronic metabolic conditions are shown as representative contexts favoring metabolic licensing and restriction, respectively. However, these outcomes are context dependent and do not represent fixed endpoints. The same stress-sensing pathways can support divergent functional outcomes depending on cell identity, signal intensity and duration, tumor niche, disease stage, and prior activation history.

### 5.1 Hypoxia and nutrient sensing (HIF-1α, mTOR, AMPK)

Hypoxia is a common feature of solid tumors and can reduce the efficacy of chemotherapy, radiotherapy, and immunotherapy. Rapid tumor growth and abnormal vascularization create regions of chronic oxygen deprivation, activating hypoxia-responsive pathways centered on HIF-1α, a major regulator of hypoxic adaptation in tumor and immune cells (124).

The effects of HIF-1α depend on cell type, signal duration, and nutrient availability. In macrophages, transient HIF-1α activation can increase glycolytic flux and support inflammatory M1-like programs when sufficient metabolic substrates remain available (125). In contrast, chronic tumor hypoxia promotes the recruitment and persistence of TAMs and MDSCs through chemokines and growth factors such as CCL26, G-CSF, and IL-6 (124, 126). HIF-1α can also increase the expression of immunosuppressive mediators, including PD-L1, ARG1, and nitric oxide-producing enzymes (127–129).

mTOR integrates growth-factor, amino-acid, and energy signals to regulate glucose uptake, protein synthesis, and glycolytic activity. In activated NK cells, mTORC1-dependent metabolic reprogramming supports increased glucose uptake and glycolysis required for effector function (27). In hypoxic human NK cells, mTORC1 can also contribute to cytokine-induced glycolytic adaptation, although this response depends on the activation and metabolic context (130). In macrophages, hypoxic TAMs upregulate REDD1, which suppresses mTOR-dependent glucose uptake and glycolysis (131). These observations illustrate that the consequences of mTOR signaling under metabolic stress vary with cell type and activation state rather than uniformly promoting either licensing or restriction.

AMP-activated protein kinase (AMPK) senses energetic stress by monitoring cellular AMP/ATP ratios and generally promotes catabolic pathways and mitochondrial maintenance (132). In macrophages, AMPK signaling has been associated with reduced NF-κB-dependent inflammatory activation and with metabolic programs linked to more oxidative, M2-like states (133). Under prolonged nutrient limitation, AMPK-mediated adaptation may favor energy conservation over sustained inflammatory activation.

Together, HIF-1α, mTOR, and AMPK integrate oxygen, nutrient, and energy status to regulate innate immune metabolism. Their combined activity influences whether innate immune cells maintain glycolytic and mitochondrial programs that support antitumor function or adapt to chronic stress by acquiring dysfunctional or immunosuppressive states.

### 5.2 Oxidative and organelle stress signaling (ROS, ER stress, UPR)

ROS are generated during mitochondrial respiration, fatty acid oxidation, and diverse signaling cascades, and serve as key metabolic stress signals linking mitochondrial dysfunction to innate immune reprogramming (134). Acute ROS bursts can cooperate with NF-κB and HIF-1α to promote glycolytic shifts and M1-like macrophage activation, licensing inflammatory effector responses needed for pathogen or tumor control. In the chronically stressed TME (134), however, sustained ROS production from dysfunctional mitochondria and high FAO contributes to TAM polarization toward M2-like phenotypes and impairs antitumor innate functions (135).

Elevated intracellular ROS also induce ER stress by perturbing ER-resident calcium channels and protein folding capacity (136). ER stress activates the unfolded protein response (UPR), which modulates cytokine production, lipid metabolism, and antigen presentation in innate immune cells. In macrophages, TME-derived cytokines such as IL-4, IL-6, and IL-10, together with uptake of oxidized low-density lipoprotein (oxLDL) via CD36 (137), engage the IRE1α–XBP1 axis and reprogram cells toward protumoral phenotypes. Studies in human colorectal cancer have shown that XBP1 activation promotes differentiation of macrophages into immunosuppressive TAMs, reinforcing metabolic restriction of inflammatory programs (138).

Thus, ROS and ER stress–UPR signaling connect oxidative and organelle stress to innate immune-cell function. Transient activation can support inflammatory responses, whereas persistent signaling in the TME promotes dysfunctional, tolerant, or tumor-supportive states.

### 5.3 Innate inflammatory sensing pathways (inflammasome, cGAS-STING, TLRs)

Environmental stressors such as cytotoxic therapies, immune effector attacks, and hypoxia induce tumor cell death and release damage-associated molecular patterns (DAMPs) into the TME. These DAMPs are detected by pattern-recognition receptors (PRRs), including TLRs, inflammasomes, and cGAS–STING pathways, which not only initiate inflammatory transcriptional programs but also reshape cellular metabolism in innate immune cells (139).

TLRs expressed on the plasma and endosomal membranes recognize DAMPs derived from necrotic cancer cells, such as heat shock proteins, extracellular ATP, and nucleic acids, and drive chronic inflammation in tumors (140, 141). In macrophages, TLR stimulation promotes a metabolic switch from OXPHOS to glycolysis via MyD88–NF-κB/mTOR signaling, licensing M1-like polarization and antitumor effector functions when nutrient support is adequate (44, 142). However, repeated or sustained TLR engagement in nutrient-limited, hypoxic niches can contribute to maladaptive inflammation and reconstitution of tumor-supportive microenvironment with TAM and tolerogenic DCs, reflecting the dual nature of TLRs as both activators and stress amplifiers (142–144).

Cytosolic sensors such as NLRP3 and cGAS–STING integrate mitochondrial damage, ROS, and metabolic cues with inflammatory cytokine production. The NLRP3 inflammasome assembles in response to PAMPs and DAMPs, activating caspase-1 and promoting secretion of IL-1β and IL-18. Under TLR2 signaling, hexokinase-driven glycolysis enhances NLRP3 activation, directly linking glucose flux to inflammasome-dependent inflammatory licensing. In certain tumor settings, coordinated TLR2/NF-κB/NLRP3 activity drives TAMs toward M1-like phenotypes and supports antitumor immunity (145). Conversely, chronic inflammasome signaling in metabolically stressed tissues can also contribute to tissue damage, fibrosis, and immune tolerance, reinforcing restriction rather than sustained effector licensing.

The cGAS–STING pathway is activated by tumor-derived cytosolic DNA and induces type I interferon production in innate immune cells, including dendritic cells (146). In DCs, cGAS–STING activation can enhance antigen presentation and T-cell priming, thereby supporting antitumor immunity (147). Although cGAS–STING can intersect with cellular metabolic and stress-response pathways, direct evidence establishing it as a broadly conserved metabolic sensor across innate immune populations remains limited.

Overall, innate inflammatory pathways act as metabolic stress sensors by coupling danger recognition to specific metabolic modules—primarily glycolysis and mitochondrial activity—that can either license or limit innate effector responses depending on the intensity and duration of signals and the underlying metabolic landscape.

### 5.4 Linking stress sensing to innate immune fate

Although HIF-1α, mTOR, AMPK, ROS, ER stress, and innate inflammatory pathways are often studied separately, these pathways can interact to integrate metabolic and inflammatory cues within the TME and shape innate immune function.

Acute engagement of these sensors can license antitumor innate immunity: hypoxia-induced HIF-1α and mTOR activation promote glycolytic programs in NK cells and macrophages (26, 148); transient ROS bursts and TLR signaling drive M1-like polarization and support DC activation (134); and inflammasome and cGAS–STING pathways reinforce inflammatory circuits when metabolic resources suffice (145). In these contexts, stress sensing enhances glycolytic engagement, mitochondrial fitness, and amino acid–supported anaplerosis, enabling robust innate effector functions (**Figure 5B**).

Under chronic metabolic stress, however, the same sensors impose restriction. Persistent hypoxia and HIF-1α signaling suppress mTOR activity and favor M2-like TAMs and MDSCs (124, 131); sustained ROS and ER stress engage UPR pathways that promote tolerogenic or protumoral macrophage states (137); adenosine and lactate accumulation dampen licensing programs in NK cells and DCs (93, 98), and prolonged inflammatory sensing can drive dysfunction rather than continued activation (**Figure 5C**).

Together, these observations indicate that innate immune fate is not determined by a single stress sensor. Rather, it reflects the integrated effects of signal duration, intensity, cellular identity, and metabolic context. These factors determine whether innate immune cells maintain antitumor function or acquire dysfunctional, tolerant, or suppressive phenotypes.

## 6 Therapeutic targeting of innate immune metabolic fate

Metabolic interventions can influence innate immune responses in tumors through two broad strategies: reinforcing metabolic licensing in effector innate cells and relieving metabolic restriction imposed by the TME and suppressive immune populations. The first approach aims to preserve glycolytic competence, mitochondrial fitness, and biosynthetic capacity in NK cells, DCs, and inflammatory macrophages. The second seeks to disrupt the metabolic programs that support TAMs, MDSCs, and TANs, or to reduce the environmental stresses that favor their persistence.

### 6.1 Reinforcing metabolic licensing in effector innate cells

Innate immune cells are central mediators of antitumor immunity, and their effector functions can be therapeutically strengthened by restoring metabolic fitness under tumor-associated metabolic stress. Metabolic fitness in NK cells and other effector innate populations can be conceptualized as a form of metabolic licensing that encompasses glycolytic competence, mitochondrial integrity, and biosynthetic readiness required for sustained cytotoxicity and cytokine production in nutrient-limited TMEs.

One major strategy is cytokine-mediated metabolic priming, which enhances nutrient uptake and energy-generating pathways to sustain effector responses. Cytokines such as IL-12, IL-15 and IL-21 can modulate NK cell metabolism (26). In human NK cells, IL-15 activates mTOR signaling and increases glycolysis and OXPHOS, supporting enhanced cytotoxic function (149). In a clinical study, the IL-15 superagonist complex ALT-803 promoted NK cell expansion and activation in patients with hematological malignancies who relapsed after allogenic hematopoietic cell transplantation (150). IL-21 has also been shown to restore metabolic fitness in dysfunctional NK cells through LDHA-dependent glycolysis and to enhance antitumor activity in solid tumor bearing mouse models (28). Combinatorial stimulation with IL-12, IL-15, and IL-18 can induce cytokine-induced memory-like NK states characterized by rapid recall responses, increased glycolytic flux, and SREBP-dependent metabolic remodeling (151, 152). However, excessive or prolonged cytokine stimulation may impair immune-cell metabolic fitness and effector function, emphasizing the need to optimize cytokine dose, exposure duration, and treatment context (153). Related metabolic adaptations have also been described in myeloid cells. In primarily non-tumor trained-immunity models, monocytes and macrophages exposed to IL-1β or β-glucan undergo Akt/mTOR/HIF-1α-dependent metabolic reprogramming associated with enhanced inflammatory responses (154–157). These studies provide mechanistic support for metabolic priming but do not directly establish therapeutic efficacy in the TME.

A second strategy is direct metabolic rewiring to stabilize energy production and functional plasticity under TME stress. Small-molecule inhibitors or activators targeting metabolic enzymes can boost effector cell fitness without relying solely on cytokines. For example, modulating aldose reductase activity or itaconate-producing pathways has been shown to reprogram NK cells and macrophages toward more robust antitumor phenotypes by optimizing glycolytic and mitochondrial metabolism and redirecting PPP activity (158–160). Treatment with aldose reductase inhibitor fidarestat reduced aldo-keto reductase family 1 member B10 (AKR1B10) expression and enhanced glycolysis in NK cells, thereby promoting NK cell effector functions in a preclinical hepatocellular carcinoma (HCC) (159). In macrophages, the IRG1-itaconate axis modulates pentose phosphate pathway activity through Glucose-6- phosphate dehydrogenase (G6PD), providing a metabolic mechanism by which macrophage inflammatory state can be reprogrammed in preclinical tumor model (158, 160). Collectively, these preclinical studies suggest that metabolic priming and targeted rewiring can reinforce antitumor immune function. However, the therapeutic relevance of these approaches depends on cellular selectivity as the same metabolic pathways may support distinct or opposing functions in tumor and immune cell compartment.

### 6.2 Reprogramming or depleting suppressive innate populations

TAMs and MDSCs represent the major immunosuppressive populations within the tumor microenvironment (161). These cells establish metabolic restriction by consuming essential nutrients, sequestering lipids, and producing inhibitory metabolites that limit effector immune cell functions. Therapeutic strategies therefore aim either to reprogram their metabolism away from suppressive states or to deplete them when reprogramming is insufficient.

One approach is to interrupt metabolic programs that sustain suppressive polarization. M2-like TAMs depend on FAO and glucose metabolism to maintain protumoral phenotypes, making these pathways key points of intervention. Pharmacological inhibition of FAO with etomoxir reduces STAT6 phosphorylation and inhibition of glucose metabolism with 2-DG suppresses M2 polarization through AMPK-HIF-1α signaling pathway, thereby restraining TAM-mediated tumor support (110, 162). In MDSCs, targeting fatty acid transport protein 2 (FATP2) with lipofermata blocks fatty acid uptake and diminishes the suppressive activity of MDSCs, thereby loosening metabolic restriction on effector cells (163). Similarly, treatment with CB-1158, an arginase inhibitor, restores amino acid availability and reverses myeloid-mediated immunosuppression (164). However, these approaches face important challenges. For example, 2-DG has non-cell specific effects whereas etomoxir exhibit dose dependent off target activity (162, 165). Similarly, targeting shared metabolic pathways, including mTOR, HIF, lactate-related metabolism may affect both tumor and antitumor immune cells, highlighting the need for careful evaluation of cellular selectivity. Moreover, most of these metabolic interventions remain supported primarily by preclinical evidence, and their evaluation in patients remains to be established.

When suppressive metabolic restriction cannot be sufficiently rewired, direct depletion of suppressive populations may provide a complementary strategy to remove persistent immunosuppressive barriers. Anti-CSF1R treatment results in TAM depletion with tumor growth inhibition depending on the tumor model (166). Likewise, MDSCs can be selectively eliminated via targeting Death receptor 5 (DR5), a receptor of TNF-related apoptosis-inducing ligand (TRAIL), which is highly expressed on MDSCs under gastric cancer and colon cancer conditions (167). These interventions may alleviate metabolic restriction by removing cells that continuously consume nutrients and generate inhibitory metabolites, thereby improving the functional capacity of antitumor innate and adaptive immune cells.

### 6.3 Relieving TME metabolic stress (lactate, hypoxia, lipids)

Metabolic stress in the TME, including lactate accumulation, chronic hypoxia, and lipid overload—profoundly impair innate immune licensing and drive immunosuppression. Targeting these environmental stressors represents a third strategic axis for restoring immune function. Reducing lactate burden can be achieved by inhibiting monocarboxylate transporters (MCTs) or glycolytic enzymes such as LDHA and PFKFB3. Blocking lactate export or production decreases extracellular acidification, improves T cell and NK cell function, and reduces the frequency of suppressive myeloid cells (168–174). Consistent with these observations, LDHA inhibitor ML-05 inhibited cell proliferation while siRNA-mediated inhibition of MCT4 in urothelial carcinoma reduced tumor growth both *in vitro* and *in vivo*, accompanied by increased tumor apoptosis (171, 174). In solid tumor models, combination treatment with MCT1 inhibitor, AZD3965 and radiotherapy significantly improved anti-tumor function (169, 172). Moreover, targeting hypoxia through hypoxia-activated prodrugs or HIF pathway modulators can selectively damage hypoxic tumor cells while limiting hypoxia-driven recruitment and maintenance of suppressive innate populations. For example, ICT10336 is a hypoxia-activated prodrug designed to release the ATR inhibitor AZD6738 under hypoxic conditions, thereby preferentially increasing DNA damage and tumor-cell death in hypoxic tumor regions (175). By attenuating HIF-dependent metabolic adaptations, these agents reduce angiogenesis, alter nutrient gradients, and partially relieve metabolic restriction on infiltrating immune cells (175, 176). Lastly, targeting lipid metabolism can enhance antitumor immunity in the TME. PLT012, a humanized anti-CD36 IgG4 antibody, binds to the fatty acid transporter CD36 and disrupts lipid uptake-driven metabolic programming in both tumor cells and immunosuppressive myeloid cells, resulting in reduced tumor progression and improved antitumor immunity in HCC (177). Together, strategies that decrease lactate, hypoxia, and lipid overload act at the level of the microenvironment to relax metabolic constraints on innate immune licensing and enhance overall immune fitness. Nevertheless, because these pathways are also required by tumor and effector immune cells, their therapeutic benefit will depend on achieving sufficient cellular and contextual selectivity.

### 6.4 Combining metabolic interventions with cancer immunotherapies

Because effective antitumor immunity requires both reinforcement of metabolic licensing in effector cells and attenuation of metabolic restriction in the TME, metabolic interventions may be particularly useful when combined with other cancer immunotherapies (178). Metabolic restriction in the TME can limit the persistence and effector function of immune cells despite checkpoint blockade, whereas restoring metabolic fitness may enhance the capacity of checkpoint-responsive immune cells to sustain antitumor activity (179). Conversely, inhibitory checkpoint signaling can further impair metabolic fitness, including glycolytic and mitochondrial function, thereby reinforcing metabolic restriction. These observations suggest that metabolic state can influence the effectiveness of immune checkpoint blockade and provide a rationale for combining checkpoint inhibitors with interventions that relieve metabolic restriction. Dietary or pharmacologic modulation of nutrient-sensing pathways can skew macrophage polarization toward M1-like states while reducing M2-associated mTOR, Nrf2, and PI3K–Akt activity (180, 181). In preclinical models, combining nutrient restriction or metabolic reprogramming with anti-PD-1 or anti-PD-L1 therapy significantly reduced tumor burden and prolonged survival, in part by decreasing immunosuppressive myeloid populations and improving effector cell licensing (168, 181). Similarly, cytokine-induced metabolic priming of NK cells enhances the efficacy of NK-based therapies, and CAR-NK approaches further amplify tumor targeting and cytotoxicity when built on metabolically competent NK platforms (182).

These examples suggest that metabolic interventions may complement checkpoint blockade, adoptive cell therapy, and other immunotherapies by supporting effector-cell metabolism or reducing metabolic barriers within the TME. To date, these combination strategies remain largely at the preclinical stage, and their safety and clinical efficacy requires further validation.

### 6.5 Challenges, context dependence, and biomarkers for stratification

Despite increasing preclinical evidence and limited clinical experience, several challenges remain. The TME is highly heterogeneous, composed of diverse stromal, vascular, and immune cell populations that differ across tumor types and even between lesions within the same patient (183, 184). Innate immune composition and metabolic wiring vary substantially, making the impact of a given metabolic intervention context dependent.

To optimize therapeutic strategies, integrated metabolic and immune biomarkers are needed for patient stratification. Measures of lactate accumulation, hypoxia signatures, and lipid metabolism can help define the metabolic state of the TME, while expression of transporters and enzymes such as MCT4 or CD36 may indicate specific metabolic dependencies (174, 177). Immune-focused biomarkers, including the abundance and metabolic profiles of TAMs, MDSCs, TANs, and effector NK cells, can further refine selection of licensing-enhancing or restriction-relieving interventions. Because metabolic pathways are shared across tumor cells, suppressive myeloid populations, and antitumor effector cells, therapeutic strategies should preferentially target metabolic dependencies enriched in specific cellular compartments rather than broadly inhibiting pathways shared across the TME (165). Ultimately, successful implementation of metabolic therapies will require matching strategies to the dominant metabolic constraints and innate immune architecture of each tumor, using biomarkers to guide combinations that reinforce antitumor licensing while dismantling key axes of metabolic restriction (**Table 1**).

**Table 1:** Therapeutic strategies that enhance metabolic licensing or alleviate metabolic restriction in innate immune cells.

Strategy	Molecular target/agent	Cellular compartment	Tumor model/cancer type	Species	Study design/development stage	Direct innate-immune endpoint	Efficacy outcome	Key limitation	Ref
Reinforcing metabolic licensing	IL-15R /ALT-803	NK cells	Hematologic malignancies	Human	Clinical study, Phase I/II (NCT01885897)	Promoted NK-cell expansion and activation	Clinical antitumor activity was observed	Systemic cytokine effects	(150)
	IL-12R, IL-15R, IL-18R/ IL-12, IL-15, IL-18	NK cells	Ovarian cancers	Human /Mouse	Preclinical	Increased glycolytic and oxidative metabolism and enhanced recall responses	Enhanced NK-cell effector function	Effects depend on cytokine dose, exposure and patient’s lipid metabolic state	(151, 152)
	IL-21R /IL-21	NK cells	MHC class I-deficient solid tumor models	Human /Mouse	Preclinical	Enhanced LDHA-dependent glycolysis in NK cells	Increased NK-cell antitumor activity	Limited tumor models and long-term metabolic effects need further validation	(28)
	IL-1R, Dectin-1 /IL-1β, β-glucan	Monocytes /Macrophages	Lung metastasis models	Human /Mouse	Preclinical	Induced Akt/mTOR/HIF-1α-dependent glycolysis	Enhanced inflammatory function	Memory effects need further validation	(154–157)
	AKR1B10 /Fidarestat	NK cells	Hepatocellular carcinoma models	Mouse	Preclinical	Promoted glycolysis and reduced AKR1B10 expression	Enhanced NK-cell effector function and reduced tumor growth	Mechanism underlying the inhibition of metastasis needs further validation	(159)
	G6PD /Itaconate	Macrophages	Lung tumor models	Human /Mouse	Preclinical	Reduced PPP activity through G6PD inhibition	Modulated macrophage inflammatory phenotype	Cellular source of itaconate requires further spatial validation	(158, 160)
Reprogramming suppressive populations	CPT1 /Etomoxir	TAMs	Several tumor models	Mouse	Preclinical	Reduced FAO-associated STAT6 signaling	Reduced M2-like polarization and tumor-supportive activity	Dose-dependent off-target effects	(110)
	Hexokinase /2-DG	TAMs	Several tumor models	Mouse	Preclinical	Suppressed glucose metabolism and M2-like polarization through AMPK-HIF-1α signaling	Reduced tumor-supportive macrophage activity	Non-cell-specific metabolic effects	(162)
	FATP2 /Lipofermata	MDSCs	Melanoma and lung tumor models	Mouse	Preclinical	Reduced FATP2-dependent fatty acid uptake	Reduced MDSC-mediated immune suppression	Tumor-specificity needs further validation	(163)
	Arginase /CB-1158	Myeloid cells	Several tumor models	Human/Mouse	Preclinical; subsequently entered Phase I clinical evaluation (NCT02903914)	Inhibited arginase activity and relieved myeloid-mediated arginine depletion	Preclinical antitumor activity and reversal of myeloid-mediated immunosuppression	Long-term safety requires further validation	(164, 185)
Depletion	CSF1R /Anti-CSF1R	TAMs	Solid tumors	Human /Mouse	Preclinical	Reduced TAM abundance	Tumor growth inhibition	Effects are dependent on treatment timing and tumor model	(166)
	DR5 /anti-DR5	MDSCs	Gastric and colon cancer models	Mouse	Preclinical	Induced MDSC apoptosis and depletion	Reduced MDSC-mediated immunosuppression	Efficacy needs further validation in humanized models	(167)
TME metabolic stress	MCT4 /MCT4 siRNA	Tumor cells	Bladder cancer models	Mouse	Preclinical	Not directly assessed	Reduced extracellular acidification and tumor growth	Direct innate-immune effects were not assessed	(174)
	MCT1 /AZD3965	Tumor cells	Lung and gastric cancer	Human /Mouse	Clinical study, Phase I (NCT01791595)	Not directly assessed	Suppressed tumor proliferation	Potential on-target toxicity and limited therapeutic window	(169, 172, 173)
	LDHA /ML-05	Tumor cells	Tumor models	Mouse	Preclinical	Not directly assessed	Suppressed tumor proliferation	Impact on immune activation needs further investigation	(171)
	ATR /AZD6738	Hypoxic tumor cells	Breast cancer models	Mouse	Preclinical	Not directly assessed	Increased death of hypoxic tumor cells	Potential toxicity to normal cells	(175)
	CD36 /PLT012	CD36-expressing tumor and immune cells	Hepatocellular carcinoma models	Human /Mouse /Primate	Preclinical; subsequently entered Phase I clinical evaluation (NCT07337525)	Reduced CD36-dependent lipid uptake and altered immune-cell metabolic programming	Preclinical antitumor immunity and enhanced tumor control	CD36-mediated lipid changes require further validation	(177)
Combination	MCT4 and PD-L1/MCT4 inhibitor + anti-PD-L1	Tumor and immune cells	Solid tumor models	Mouse	Preclinical	Improved immune infiltration and effector functions	Improved tumor control	Clinical translatability remains to be established	(168)
	Multiple/ Cytokine priming + CAR-NK	NK cells	Lymphoma	Human /Mouse	Preclinical	Enhanced NK-cell metabolic fitness and cytotoxicity	Improved tumor targeting	Persistence, safety, and clinical efficacy need further validation	(182)

## 7 Conclusion and future directions

Tumor progression is accompanied by metabolic remodeling that reshapes the function and fate of innate immune cells within the tumor microenvironment. As discussed throughout this review, nutrient availability, oxygen tension, and tumor-derived metabolites influence innate immune activation, persistence, dysfunction, and suppressive reprogramming. Metabolic pathways therefore do more than supply energy and biosynthetic intermediates; they influence whether innate immune cells can acquire and maintain effective functional states. The concepts of metabolic licensing and metabolic restriction offer a useful way to understand these context-dependent outcomes. Metabolic licensing supports antitumor function through glycolytic engagement, mitochondrial fitness, amino acid availability, and cytokine-driven metabolic priming, whereas chronic metabolic stress can limit effector activity and favor dysfunctional, tolerant, or immunosuppressive states.

New experimental approaches are making it possible to examine innate immunometabolism with greater cellular and spatial resolution. Single-cell and spatial metabolomics, imaging mass spectrometry, CRISPR-based functional screening, and dynamic metabolic tracing can reveal how metabolic states differ across immune populations and tumor regions. Combining these approaches with phenotypic and spatial information should help define how innate immune cells acquire, maintain, or lose metabolic fitness within tumors.

Several questions remain open. Metabolic licensing and metabolic restriction are better viewed as context-dependent positions along a continuum rather than fixed states. The same metabolic stress can produce different outcomes in NK cells, dendritic cells, macrophages, neutrophils, and MDSCs, indicating that cell type-specific signaling and epigenetic programs shape the response. It will also be important to determine how metabolic adaptation intersects with trained immunity, tolerance, and other forms of persistent dysfunction, and whether these states can be distinguished by measurable metabolic features.

Therapeutic strategies that enhance metabolic licensing or relieve metabolic restriction may improve cancer immunotherapy. Future studies should identify biomarkers that distinguish metabolically fit from dysfunctional innate immune populations and define interventions that reprogram immune metabolism without disrupting systemic homeostasis. A better understanding of these processes may support the development of treatments that preserve antitumor innate immunity while limiting suppressive adaptation in the tumor microenvironment.

## Glossary

DC: Dendritic cell
FAO: Fatty acid oxidation
HIF: Hypoxia-inducible factor
MDSC: Myeloid-derived suppressor cell
NK: Natural killer
OXPHOS: Oxidative phosphorylation
ROS: Reactive oxygen species
TAM: Tumor-associated macrophage
TAN: Tumor-associated neutrophil
TCA: Tricarboxylic acid
TME: Tumor microenvironment
PPP: Pentose phosphate pathway
ER: Endoplasmic reticulum
NOS2: Nitric oxide synthase 2
LPS: Lipopolysaccharide
GPD2: Glycerol 3-phosphate dehydrogenase
GPS: Glycerol 3-phosphate shuttle
ECAR: Extracellular acidification rate
OCR: Oxygen consumption rate
OPA1: Optic atrophy 1
2-DG: 2-deoxy-D-glucose
NAD: Nicotinamide adenine dinucleotide
oxLDL: Oxidized low-density lipoprotein
LDH: Lactate dehydrogenase
FBP1: Fructose-1,6 bisphosphatase
GzmB: Granzyme B
